# Benchmarking Human Performance for Visual Search of Aerial Images

**DOI:** 10.3389/fpsyg.2021.733021

**Published:** 2021-12-14

**Authors:** Rebecca E. Rhodes, Hannah P. Cowley, Jay G. Huang, William Gray-Roncal, Brock A. Wester, Nathan Drenkow

**Affiliations:** Johns Hopkins University Applied Physics Laboratory, Laurel, MD, United States

**Keywords:** aerial images, visual search, human performance benchmark, scene perception, geospatial analysis, human machine teaming

## Abstract

Aerial images are frequently used in geospatial analysis to inform responses to crises and disasters but can pose unique challenges for visual search when they contain low resolution, degraded information about color, and small object sizes. Aerial image analysis is often performed by humans, but machine learning approaches are being developed to complement manual analysis. To date, however, relatively little work has explored how humans perform visual search on these tasks, and understanding this could ultimately help enable human-machine teaming. We designed a set of studies to understand what features of an aerial image make visual search difficult for humans and what strategies humans use when performing these tasks. Across two experiments, we tested human performance on a counting task with a series of aerial images and examined the influence of features such as target size, location, color, clarity, and number of targets on accuracy and search strategies. Both experiments presented trials consisting of an aerial satellite image; participants were asked to find all instances of a search template in the image. Target size was consistently a significant predictor of performance, influencing not only accuracy of selections but the order in which participants selected target instances in the trial. Experiment 2 demonstrated that the clarity of the target instance and the match between the color of the search template and the color of the target instance also predicted accuracy. Furthermore, color also predicted the order of selecting instances in the trial. These experiments establish not only a benchmark of typical human performance on visual search of aerial images but also identify several features that can influence the task difficulty level for humans. These results have implications for understanding human visual search on real-world tasks and when humans may benefit from automated approaches.

## Introduction

After the 2010 Haiti earthquake, aerial imagery facilitated damage assessment by allowing hundreds of crowdsourced workers to assess high resolution images of buildings and produce post-disaster damage maps (World Bank et al., 2010). More generally, aerial imagery has played a critical role in resolving global conflicts and aiding humanitarian efforts (Appeaning Addo, 2010; Weir, 2015; Kolak et al., 2018). Despite being commonly used to inform high-level decision making, aerial imagery analysis involves manual components and is prone to mistakes due to attentional limitations of humans. While error rates in the field of geospatial analysis may be unclear, in the field of radiology – another domain in which imagery analysis is used to inform critical decisions – false positives in disease diagnosis occur as often as 25% of the time, leading to unnecessary invasive procedures that can affect clinical outcomes (Anbil and Ricci, 2020). In the context of Haiti damage assessment, false positives could have resulted in unnecessary allocation of resources to areas that suffered less damage. Like the recent incorporation and increasing adoption of artificial intelligence into radiology, advances in machine learning and image recognition may be applied to geospatial information to complement manual analysis (Traylor, 2019; Arthur et al., 2020). Complex object recognition and visual search tasks like aerial image analysis will likely require a “human-in-the-loop” system, the goal of which will be to leverage the strengths of both humans and machines (Arthur et al., 2020). A robust understanding of how humans perform these complex visual tasks, and where they fail, will be important for building successful human-machine teams.

Object detection in aerial images is an active area of research in computer vision due to the challenges of detecting objects with massive variations in scale and orientation (Tayara and Chong, 2018; Ding et al., 2021). Given the increased prevalence of satellite-based imaging, there is a wealth of data available for training machine learning algorithms to learn the relevant information for object recognition from the data themselves. Researchers are often interested in comparing their machine learning based approaches against human performance and seek algorithms that can match or exceed human performance (Krizhevsky et al., 2017; Silver et al., 2017; Yang et al., 2019). While progress has been made to compare human and machine learning performance (Geirhos et al., 2018), there is still a need for better benchmarks of typical human performance that algorithms can be measured against. Thus, one of the goals of the present study was to establish a benchmark of typical human performance on an aerial image search task. Understanding which features of the task are likely to impair human performance could facilitate human-machine teaming by identifying which aspects of the task would benefit most from automated systems. While psychophysics experiments provide a mechanism for studying these factors under controlled settings (Schönfelder and Wichmann, 2012; Geirhos et al., 2018), understanding failure modes on real-world, application-specific datasets is equally important and requires unique considerations. A second goal of the present study was to identify features of aerial images that make performance more challenging for humans.

The ability to predict how difficult it might be for humans to identify or count targets in an aerial photograph based on its features could help inform when additional independent reviews of the image may be needed or when humans could particularly benefit from automated approaches to establish convergence. Although, research is fairly limited on human performance on scene recognition in aerial photographs, much has been learned from classic object recognition and visual search tasks as to the types of image features that influence human performance, which may also apply to real-world aerial photographs. For example, research suggests that color is an important guide of attention, leading to “pop-out” effects when a target differs in color from a homogeneous set of distractors (Egeth et al., 1972; Lloyd, 1997). In fact, color has been identified as a feature that undoubtedly guides visual search, along with size and orientation (Wolfe and Horowitz, 2017). A recent study with real-world photographs examined how several image properties correlated with the difficulty of searching for objects such as airplanes, boats, cats, dogs, etc. and found that the size of the objects was an important factor in determining human ratings of difficulty (Ionescu et al., 2017).

Aerial images have a number of other features that could influence human performance. For example, people recognize objects more easily when they appear at the size that they are used (Konkle and Oliva, 2011); however, objects in aerial photographs are non-canonical in size, and all of the objects in the image are being experienced from a viewpoint that is unfamiliar to most observers (Edelman and Bulthoff, 1992; Leek, 1998; Garsoffky et al., 2009). Lack of information and additional factors such as resolution, lighting, color, and occlusion can make object recognition and visual search in aerial images challenging (Cain, 1962; Happel et al., 2005). While humans may be able to overcome low resolution with enough knowledge of other helpful visual cues (Torralba et al., 2008; Dodge and Karam, 2017), aerial images lack certain helpful cues such as canonical size and orientation.

Natural scenes often involve searching for more than one target, but often the number of targets is unknown. Research on multiple target search also suggests that when there are an unknown number of targets, people often miss lower salience targets after identifying a high salience target (known as sequential search misses), due to errors in scanning, recognition, strategy (i.e., optimizing efficiency over accuracy), or other causes (Cain et al., 2013). This pattern has been found repeatedly in studies of search errors in radiology studies (Berbaum et al., 1990; Berbaum, 2012). Sequential search misses may be likely to occur in searches of real-world aerial images as well when the number of targets is unclear. Moreover, targets in aerial images may belong to the same class (e.g., car), but may vary widely in features like color, resolution, and occlusion, all of which may impact the salience of an object. Research suggests that when people search for multiple targets, performance is better when people are looking for similar targets and accuracy suffers when looking for search for dissimilar targets (e.g., cars of different colors, shapes, or orientations; Menneer et al., 2007). This implies that the inherent variability in natural scenes may pose a challenge for searching for multiple targets.

One model of visual search of complex scenes proposes a two-pathway process: one pathway involves recognition of individual objects, while the second pathway extracts global information from the scene (Wolfe et al., 2011). This model explains why searching for bread in a kitchen is more efficient than searching among a set of random objects, as well as why radiologists are able to rapidly extract diagnostic information from medical images (Drew et al., 2013). Similarly, models of gaze allocation in scene viewing suggest that low-level image features can predict fixations to some extent, but higher-level cognitive processes also play a role (Tatler et al., 2011). For example, some studies have found that semantic context guides visual search such that people look for a target in areas of the image where they expect to find the target based on prior knowledge (Neider and Zelinsky, 2006; Fernandes et al., 2021). In the context of aerial images, some research suggests that experts are better able to use semantic information in aerial images over low-level visual information, again suggesting a role of knowledge and context (Šikl et al., 2019). While semantic context likely plays a role in aerial images, given the unfamiliar viewpoint and shapes of objects in the scene, the average viewer may have less expectation of where to look for targets. Thus it is possible that low-level features could play a more significant role in aerial images than in more familiar scenes.

This study examined human performance on a counting task using a large set of satellite images that had previously been annotated by experts in aerial image interpretation. The task was chosen to be representative of real-world tasks, in which viewers have an image of the object of interest and must locate it in a larger image – for example, a search and rescue task where an individual must look through drone images to locate a person of interest. Experiment 1 examined the effect of target size, target location, and the number of targets in the aerial scene on performance and search strategies. Experiment 2 examined these issues further with the same type of images and also assessed the influence of color and clarity.

## Experiment 1

### Materials and Methods

#### Study Participants

We recruited 446 unique participants from Amazon Mechanical Turk (MTurk). Following best practices, inclusion criteria were set to maximize the quality of participants (Amazon Mechanical Turk, 2019). To be eligible for our study, participants needed to have completed at least 500 human interaction tasks (HITs) on MTurk, received at least a 95% approval rate for HITs completed, and use a laptop or desktop with a screen resolution of at least 1,200 × 910. In total, 29 participants (7%) were removed from analysis due to not following instructions or incorrectly answering quality monitoring trials; the remaining 417 participants were included in the analysis. On average, participants spent about 30 min on the entire task, and participants were paid $5.00 for each HIT they completed.

The experiment was conducted in three periods over 30 days in order to maximize the number of responses. Newer HITs on MTurk have greater visibility, so in each period of data collection, we re-posted the task to attract new participants. In the first two periods, participation was limited to first-time participants and they were allowed to complete only a single block of trials (i.e., one HIT). In the third period of data collection, repeat participants were allowed due to lower response rates, but participants could still only complete one block of trials, and the block consisted of different trials than the ones they saw previously. Across all periods, a total of 116 participants (28%) completed more than one block of trials. Eight participants were erroneously assigned the same block twice, and in these cases only responses from the first block were included. Since each participant saw different trials in each block and they did not receive any feedback on the correctness of their answers, we did not expect that previous experience on the task would substantially affect performance.

#### Aerial Image Stimuli

500 trials were created from images selected from the Dataset of Object deTection in Aerial images (DOTA) dataset (Xia et al., 2018), which consisted of 2,806 satellite images from Google Earth containing 15 distinct object classes as shown in Table 1. Annotations were obtained from the iSAID dataset (Zamir et al., 2019), which included precise and valid annotations by expert annotators for the images in the original DOTA dataset. The iSAID and DOTA datasets are unique given that the images are of high spatial resolution, contain a variety of common object categories with a large number of instances, and realistic variation in object scale and orientation. We omitted two classes (i.e., “harbor” and “bridge”) from our trials due to overlap with the “ship” and “vehicle” classes; it would have been difficult to present a target image of a bridge or harbor that did not include a vehicle or ship, respectively, which would have caused confusion about which object class participants should search for.

**Table 1:** Number of trials for each search template class in Experiment 1.

Search template class	Number of trials
Baseball diamond	39
Basketball court	39
Ground track field	37
Helicopter	30
Large vehicle	32
Plane	41
Roundabout	33
Ship	43
Small vehicle	53
Soccer ball field	32
Storage tank	45
Swimming pool	40
Tennis court	36
Harbor	NA
Bridge	NA

#### Study Procedure

The primary task asked participants to search a large scene and place a marker on all objects that were the same type as the search template. A single HIT on MTurk contained one block of trials, which consisted of three training trials followed by 20 experimental trials and three quality monitoring trials to help ensure and validate participant engagement and attention. Each HIT presented a link which, when clicked, would direct participants to one of 25 different possible blocks. All participants in a block saw the same experimental trials in a random order. At the end of the HIT, participants completed a post-task survey providing feedback on the perceived difficulty, enjoyability, and other dimensions of the task. Details on the training trials, quality monitoring trials, and post-task survey can be found in the Supplementary Material. On average, 20–21 participants completed each HIT.

#### Experimental Trials

**Figure 1:**
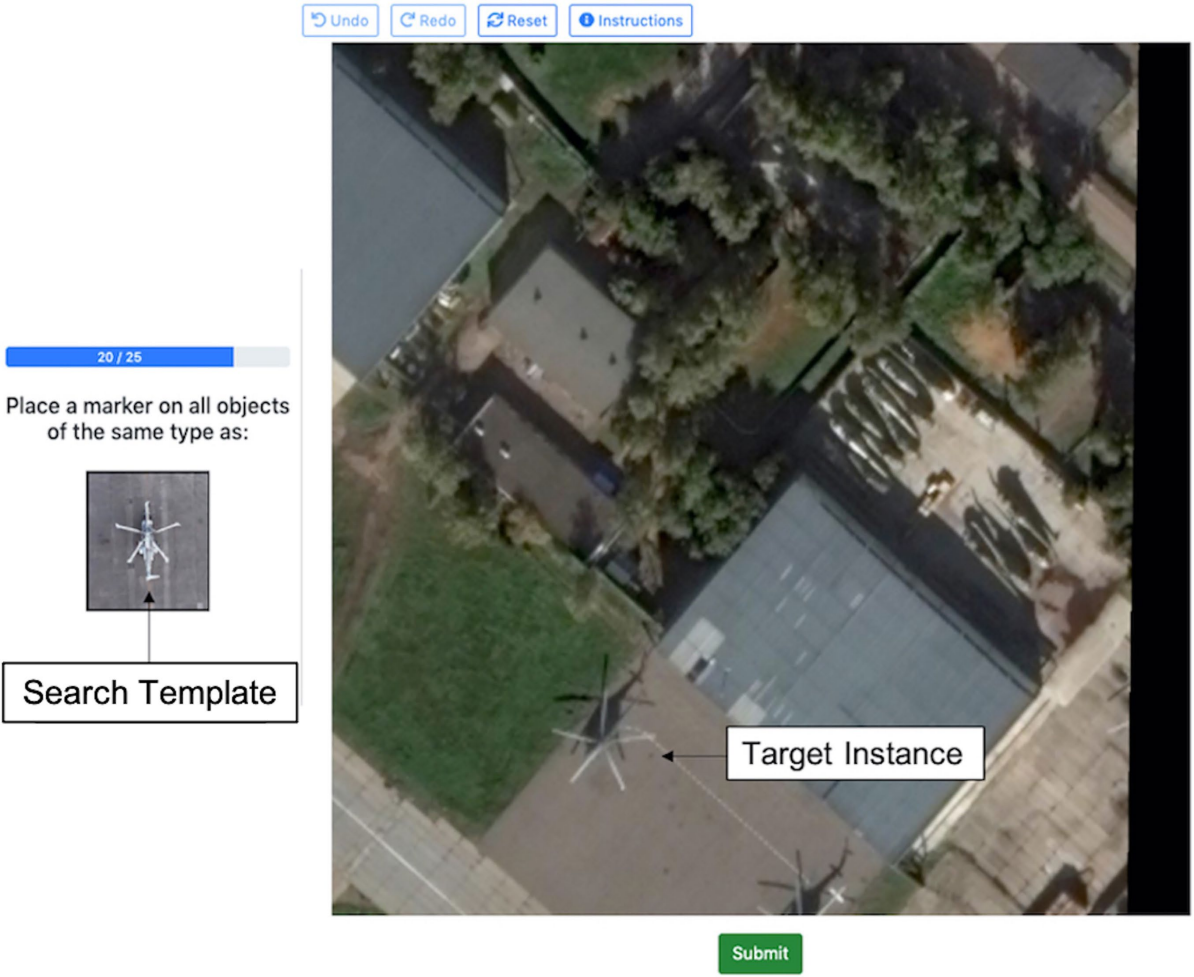
Annotated example of an experimental trial in Experiment 1. The helicopter shown in the thumbnail target image is the “search template” and the helicopter shown in the larger search image is a “target instance.” Image provided by iSAID dataset (Zamir et al., 2019) using Google Earth images.

Each experimental trial presented the participant with a matching scenario and participants were asked to identify instances of the search template within an aerial image. Each trial consisted of a search template thumbnail (containing the object class of interest) and a larger aerial search image, in which to count target instances. Participants were told to search this scene for “objects of the same type” as the object shown in the search template (Figure 1). Ground truth target instances were defined by the expert annotations provided in the iSAID dataset; participants’ selections were scored based on whether they matched these ground truth annotations. The exact search template was not actually present in the scene shown in the search image; instead, the search template was sampled from a different scene that also contained that object class. Therefore, the search template did not exactly match any portion of the search scene.

Search images were sampled to always contain at least one target instance and were limited to a maximum of 30 target instances. The number of target instances in each trial ranged from 1 to 25; most of the trials (70%) had only one or two target instances, 27% had between 3 and 10 target instances, and only 3% had more than 10 target instances. Participants were not required to select an object to advance to the next image.

The search template remained visible for the duration of the trial. The instructions were left intentionally vague about what constituted an exact match to the search template. For example, if the target image was a sedan, participants would have to decide whether a van belonged to the same class. The ambiguity was intended to emulate real-world search tasks, in which the features that define target classes may be ambiguous. Furthermore, we were interested in identifying features that impacted judgments about matches. There was no magnification feature in the scene, so participants could not zoom into particular parts of the image to view them more closely; participants were, however, free to use the zoom feature on their browser. The decision to prevent magnification was again driven by a desire to mimic real-world searches of aerial photographs for which magnification is often not available. We also aimed to mimic the amount of information that is ordinarily available to machine learning algorithms, which typically do not use magnification features.

#### Identifying Correct Matches

For each object that a participant selected, the x-y coordinates of the selection were recorded as well as the timestamp. The x-y coordinates of each selection were compared with the x-y coordinates of the ground truth locations of the target instances in the search image. If a participant’s selection matched the true location of a target instance, that selection was considered a true positive. If a participant selected an object that was not a target instance, that selection was considered a false positive. Target instances that were *not* selected were considered false negatives. True negative rates were not calculated, since there was not a discrete number of negative instances in the search image; rather, any area of the image that did not contain a target instance and was left unselected by participants would be considered a true negative.

Matching was performed using the Munkres algorithm, which matches ground truth target instance locations and participant-selected locations based upon minimizing pairwise Euclidean distance between the matched locations (Kuhn, 1955). Since the Munkres algorithm finds a matching regardless of the absolute distance between match locations, an additional filtering step was added to discard matches that were separated by a distance greater than the average dimension of the target instances in the search image. Since the overall altitude of the camera varied from image to image, the filtering threshold was determined per image per target class. We used a radius that was equal to the average dimension of the bounding boxes because using the average gave us more consistency across all instances in the image (which might have highly variable dimensions even within a single image). This also roughly simulated the margin of error for clicking on the correct instance. Since the ground truth point was used as the center of the true bounding box, clicking within the radius described would approximate clicking somewhere within the true box.

#### Analyses

We calculated the true positive, false negative, and false discovery rates across all participants and trials. The true positive rate was calculated as the proportion of true target instances that were correctly identified as targets (i.e., $\frac{TP}{TP+FN}$). The false negative rate was calculated as 1 – TP, and the false discovery rate was calculated as the proportion of selections that were false positives (i.e., $\frac{FP}{FP+TP}$).

Regression analyses examined the influence of the size and location of the true target instances, as well as the number of true target instances in the search image, on performance. We expected that performance would be better on trials that had larger target instances, since these are likely more noticeable and size is known to be an important feature that guides attention in visual search. We also expected that targets that were closer to the center of the image may be noticed more easily and thus increase performance, and that having more targets present in an image would result in more missed targets. To analyze the impact of location, we calculated the Euclidean distance between each target instance and the center of the image (*X* = 400, *Y* = 400). To understand the spatial distribution of participants’ selections as well as the distribution of true target instances, we divided the search image into a 3 × 3 grid. Since the image was 800 × 800 pixels, each grid cell was 266 × 266 pixels. The grid cells were numbered horizontally from left to right, beginning with the bottom left corner of the image.

Additional analyses examined the relationship between the number of targets and the likelihood of selecting objects or missing targets, to assess when participants tended to give up looking for targets. We also examined selection order within a given trial to understand what features affected the order in which participants selected target instances in the image, which may be used as a proxy for their salience. We expected that, on average, larger target instances would be selected before smaller target instances.

### Results

On a given trial, the hypothetical worst-case performance would occur if participants only selected objects that were distractors and failed to select any of the true target instances, which would result in 0% true positives, 100% false negatives, and a 100% false discovery rate. Across all trials in this task, the true positive rate was 68% and the false negative rate was 32%, indicating that participants correctly identified target instances as targets most of the time but still missed many targets. The false discovery rate was 34%, indicating that participants often found more targets than there actually were in the image. The median time to complete a trial was 7.85 s (mean = 16.74 s). The median time of the first selection was 5.03 s (mean = 12.94) and the median time participants took between selections was 1.02 s (mean = 1.97 s).

#### Relationship Between Selections and Number of Targets

**Figure 2:**
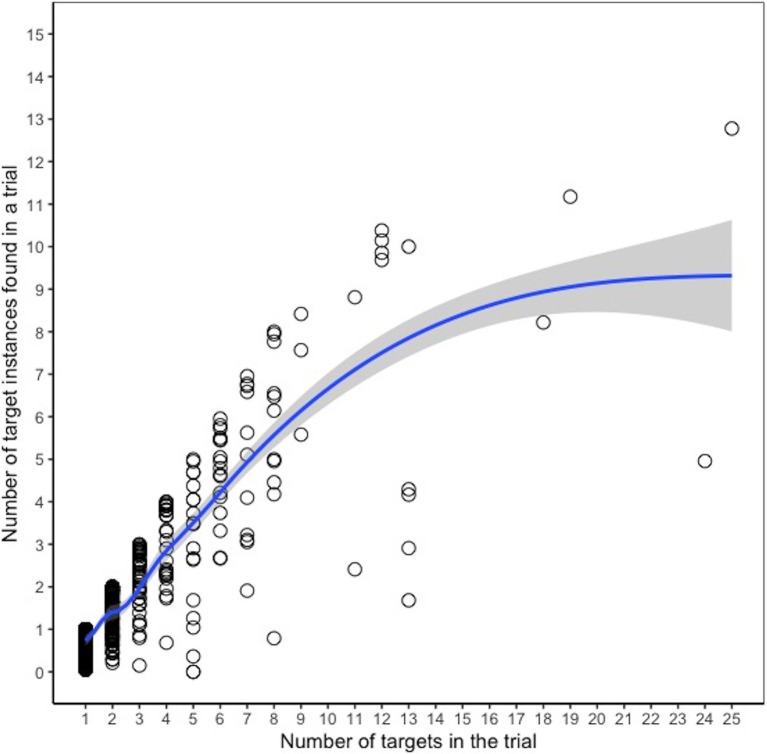
Number of targets found in a trial vs. number of targets in the trial, Experiment 1. Each dot represents a given trial, and the *y*-axis indicates the average number of targets found (across participants) in that trial. The line is a loess curve.

In 6% of trials, participants submitted it blank without selecting any objects. Among trials where an object (not necessarily a target instance) was selected, the number in each trial ranged from 1 to 70, with a median of 2 (mean = 2.93). Among these trials, participants selected only 1–2 objects in the search image for most of the trials (64%) and 3–10 objects in 31% of the trials. Fewer than 3% of these trials had more than 10 objects selected. We expected that the probability of missing a target would increase as the number of targets in the image increased. To test this, we calculated the correlation between the false negative rate for a trial and the number of targets in the trial and found a significant (albeit weak) positive relationship, suggesting the rate of false negatives increased slightly with more targets, *r*(494) = 0.12, *p* < 0.05. There was also a significant negative relationship between the false discovery rate and number of targets in a trial [*r*(494) = −0.31, *p* < 0.001], indicating that there were more false positive selections when there were few target instances in the trial. Figure 2 shows a scatterplot and loess curve showing the relationship between the number of targets in a trial and the average number of target instances found, where each data point represents a trial. Since most trials only had 1–2 target instances, it is difficult to identify the point at which performance levels off. However, the figure suggests that the number of target instances found was generally linearly related to the number of targets for trials with about 10 or fewer targets. While there are sparse data for trials with more than 10 targets, the graph suggests that the ability to find additional targets began to level off after that point.

#### Instance-Level Model Predicting Target Instance Selections

We constructed a multilevel logistic regression model to understand which features had an impact on the accuracy of identifying target instances, using the lme4 package in R (Bates et al., 2015). Participant and trial number were entered as random effects in a random intercepts model. The dependent variable was whether a target instance was selected or not. Thus, a value of 1 indicated a true positive selection and a value of 0 indicated a false negative or missed target instance. False positive selections were not included in this analysis, since information about object size was only available for true target instances. Since the response variable was dichotomous, a logistic regression model was used. The fixed effects included the size (area in pixels) of the target instance, the Euclidean distance from center, and the number of targets in the image. Since the size of the target instance was on a much larger scale and was heavily skewed right, with a mean of approximately 125 × 125 pixels but a median of 66 × 66 pixels, the variable was log transformed. Plots of the logit values vs. the untransformed and transformed size variables showed a more linear relationship with the transformed variable, suggesting better satisfaction of regression assumptions.

The instance-level model revealed that both the target instance size and distance from center were significant predictors of target instance selections (Table 2). Larger target instance sizes were more likely to be selected, and target instances that were located closer to the center of the image were also more likely to be selected. The number of targets in an image was a marginally significant predictor. For true positive selections, the average target size was approximately 139 × 139 pixels and the average distance from center was 299 pixels. For missed targets, the average target size was approximately 83 × 83 pixels and the average distance from center was 323 pixels. Generally, grid cell 5 (middle grid cell) appeared as one of the most frequently selected locations in the first few selections. Out of all objects selected by participants in the first two selections of a trial (*N* = 14,917), the largest percentage (15%) were located in grid cell 5, followed by grid cell 8 (top row, middle column; 13%). The spatial distribution of true target instances (*N* = 1,330) suggested that the largest percentage were located in grid cell 1 (bottom row, left column; 15%) and grid cell 2 (bottom row, middle column; 15%). Grid cell 5 contained 12% of target instances.

**Table 2:** Multilevel logistic regression model predicting accuracy from target instance features, Experiment 1.

Predictor	Estimate	SE	*z*	*p*
Intercept	−5.88	0.32	−18.02	<0.001
Log (target instance size)	0.92	0.03	29.18	<0.001
Target instance distance from center	−0.001	0.00	−5.84	<0.001
Number of target instances	−0.05	0.03	−1.71	0.08

The variance of the random effects was 3.89 for trial and 0.95 for worker ID, indicating that there was greater trial-related variance than worker-related variance. We calculated the *R*^2^ of the model following the method described in Nakagawa and Schielzeth (2013), which calculates marginal *R*^2^ (variance explained by fixed effects) as well as conditional *R*^2^ (variance explained by fixed and random effects). To estimate the relative influence of each fixed effect, we constructed a series of separate regression models that each contained the random effects and one fixed effect of interest, and compared the pseudo-*R*^2^ of each of these models to a baseline model with only random effects. Target instance location explained only 0.82% of the variance, while size explained 16% of the variance, indicating that target instance size was the more influential predictor. In the full model with all fixed effects, the fixed effects explained 16% of the variance and the combination of fixed and random effects explained about 57%.

**Figure 3:**
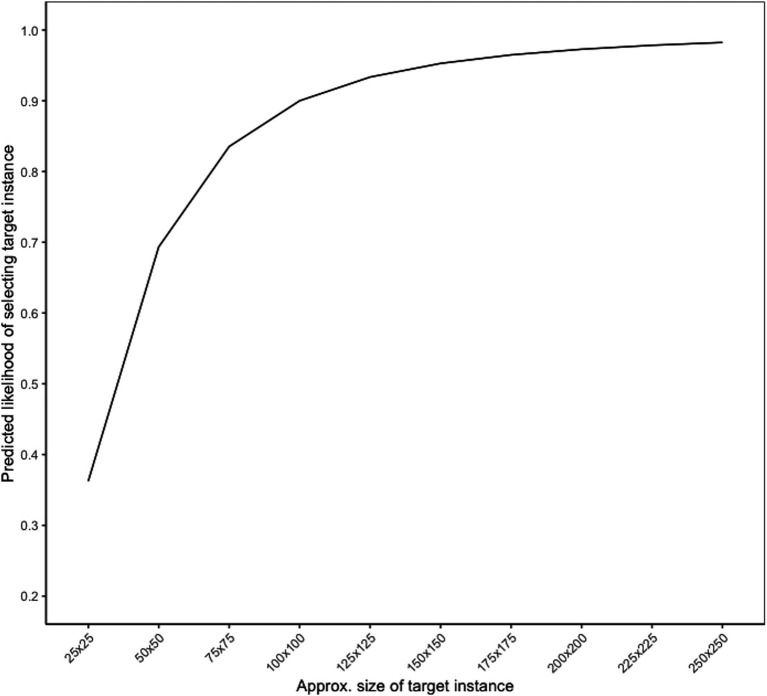
Likelihood of identifying target instance, by size.

Figure 3 shows the predicted likelihood of correctly identifying a target instance (i.e., probability of a true positive) based on its approximate size. These target sizes are approximate because they represent the square root of the total area of the target instance; for example, a true target instance size of 10,000 pixels could mean the target instance was 100 × 100 pixels or 200 × 50 pixels. For ease of interpretation, we denote the approximate target size based on the square root. In this scenario, the predicted true positive rate was above 90% when the target instance size was approximately 100 × 100 pixels or larger in total area. For reference, in the present study, the median target instance size was approximately 66 × 66 pixels in total area, indicating that half of the target instances were smaller than this and would have a predicted probability of less than 80%.

We constructed a similar multilevel regression model to predict false positive selections; in this case, the dependent variable represented whether the selection was a false positive (1) or a true positive (0). Since object size was only available for targets, this variable was not included in the model; the independent variables were distance of the selected object from the center of the image and the number of targets in the trial. Both variables were significant predictors of the likelihood of a selected object being a false positive. Specifically, the fewer the number of targets in the image, the more likely a selection would be a false positive (*B* = −0.28, *SE* = 0.04, *t* = −7.76, *p* < 0.001). Trials with only one or two targets had some of the highest false discovery rates (57 and 36%, respectively), suggesting that participants thought there were more targets on these trials than there actually were. Distance from center was negatively associated with the likelihood of a false positive selection (*B* = −0.002, *SE* = 0.0002, *t* = −8.12, *p* < 0.001), but the average distances from center for false positive and true positive selections showed only small differences (285 pixels and 289 pixels, respectively), suggesting this was not a strong effect.

#### Influence of Size on Selection Order

To better understand participants’ search strategies, we next examined how the size of the target instances influenced the order in which people selected them. We selected a subset of trials, in which participants had selected at least two target instances and we again limited the analysis to true positive selections only, since information about object size was only available for the true target instances. Additionally, we limited the number of target instance selections to a maximum of 10, since the majority of trials (96%) had 10 or fewer instances selected. With these constraints, there were 13,863 true positive selections, and each possible order of selection in their respective trials (1:10) had at least 100 data points. Specifically, there were 3,816 true positive selections that were selected first in their respective trial, and 125 true positive selections that were selected 10th in their respective trial. For each trial, we ranked the target instances that participants selected by size, with 1 representing the largest target instance in the trial. We then estimated the Spearman’s correlation between the order the target instances were selected for the first 10 selections and their ranked size within the trial. Because there were different numbers of target instances in different trials, one would expect a significant correlation with selection order even if target instances were ranked arbitrarily rather than by size. To account for this, we calculated the correlation between selection order and arbitrary ranks first as a baseline. Arbitrary ranks were calculated by generating a random number for each target instance and then ranking them from largest to smallest within each trial.

The correlation between selection order and arbitrary ranks was indeed significant (*r_s_* = 0.37, *p* < 0.001). The correlation between the selection order and the ranked size of the target instance in a trial was also significant with a larger magnitude (*r*_s_ = 0.46, *p* < 0.001). We tested whether the difference between the two correlation coefficients were statistically significant using the cocor package in R (Diedenhofen and Musch, 2015) for overlapping correlations based on dependent groups, which showed that the correlation between selection order and ranked size was significantly larger than the correlation between selection order and arbitrary ranks (Williams’ *t* = 10.88, *p* < 0.001).

**Figure 4:**
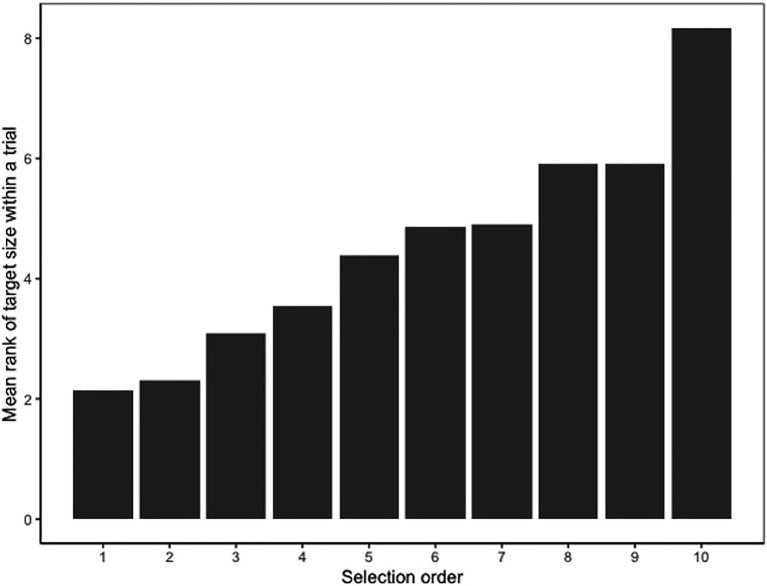
Average ranked size of target instance by selection order for Experiment 1. Lower ranks indicate larger target instances in the trial.

Figure 4 illustrates the average ranked size of a target instance by the order in which it was selected. In general, target instances selected first had lower ranks, indicating larger sizes in the trial, than target instances selected later.

### Discussion

Experiment 1 results suggested that the task of finding targets in aerial photographs poses a challenge for human participants. While participants selected correct targets most of the time, they still missed many targets and often erroneously identified non-target objects as target instances. Participants also reported a medium workload and reasonably high effort on the task. As expected, larger target instances were more likely to be selected, while smaller target instances were more likely to be missed. Larger target instances were also more likely to be selected early in a trial. Target instances that were located closer to the center of the image were more likely to be selected, suggesting that the target instances that appeared in that location were more noticeable or participants spent more time looking for targets in those areas. While the number of targets in a trial was not a significant predictor of correctly identifying target instances, it was a significant predictor of making a false positive selection in the trial, and false positives were more likely when there was a smaller number of targets. One interpretation of this finding could be that participants expected there to be more targets in these trials, leading them to mistakenly identify some objects as targets. Target instance clarity was not examined as a feature in this experiment, but qualitative feedback from participants suggested that target instance clarity likely had an influence on accuracy, as this feature was mentioned as the main determinant of the difficulty of the trial.

Experiment 1 confirmed that searching for targets in aerial photographs is not trivial for humans, and there are multiple features of these images that can lead humans to make errors. A limitation of this experiment was that there was relatively limited information available about the target instances and how they influenced performance. For example, color is known to be a guiding attribute of visual search, and target instances that match the target color may be more likely to be selected, especially in cases where there is a lack of meaningful cues from other features of the scene. Additionally, qualitative feedback from participants suggested that clarity of the search template and target instances is likely to have an influence on how people perform the task. Consequently, Experiment 2 investigated the influence of these features directly in addition to target instance size, location, and number of targets.

## Experiment 2

### Materials and Methods

#### Study Participants

We recruited 249 unique participants from Amazon Mechanical Turk, using the same qualification criteria as Experiment 1. The Experiment 2 task consisted of a single trial rather than 25 trials, so quality monitoring trials were not included. Instead, we examined the distribution of the time participants spent on the trial to identify excessively short durations. None of the participants spent less than a minute on the task, and none of the participant feedback indicated any technical or other issues with the task. Therefore, no participants were excluded from this experiment. Participants were paid $1.50 for each HIT they completed.

Experiment 2 used a subset of 200 trials from the DOTA dataset used in Experiment 1. Since participants had to answer multiple questions about each target instance in Experiment 2, and some search images had as many as 20 target instances, we limited the HIT to a single trial in order to ensure that the task would be a reasonable length for participants. We aimed to have approximately 20 responses per trial, consistent with Experiment 1; however, since each HIT consisted of a single trial, this would have required 4,000 unique participants if each participant was only allowed to complete a single trial. To reduce the number of unique participants needed, for each HIT, we allowed participants to complete multiple trials (up to 50). We conducted this experiment in four periods of data collection, with each period having 50 unique trials available. To restrict the maximum number of trials participants could complete, in each period, we restricted participation to those who had not participated in a previous period. In reality, across all data collection periods most participants (57%) completed fewer than 10 trials, and only six participants completed 50 trials. Due to a technical issue, 47 participants from Experiment 1 participated in Experiment 2. Given the fact that Experiment 2 took place several months after Experiment 1 and we did not provide any performance feedback, we did not expect prior experience with the task to have a meaningful effect on performance.

#### Stimuli

Trials were selected to have a wide range of accuracy scores to ensure variability in task difficulty. To select trials, the average true positive rate across participants from Experiment 1 was calculated for each trial. Ten performance bins were created based on the average trial-level true positive rate in increments of 10%. For each performance bin, 20 trials were randomly selected, for a total of 200 trials. Table 3 shows the number of trials associated with each search template class. On average, each HIT was completed by 19–20 participants, consistent with Experiment 1.

**Table 3:** Number of trials for each search template class in Experiment 2.

Search template class	Number of trials
Baseball diamond	17
Basketball court	15
Ground track field	23
Helicopter	10
Large vehicle	16
Plane	17
Roundabout	14
Ship	16
Small vehicle	19
Soccer ball field	12
Storage tank	16
Swimming pool	11
Tennis court	14

Similar to Experiment 1, the number of true target instances in each trial ranged from 1 to 19; 72% of trials had only 1–2 target instances, 25% had 3–10 target instances, and only 3% had more than 10 target instances. Participants were not required to select an object to complete the trial.

#### Study Procedure

In contrast to Experiment 1, each HIT consisted of only a single trial; thus, for every HIT, participants saw one search template and one aerial search image. Participants were first asked to search the aerial image for objects that matched the search template, as in Experiment 1. Afterward, participants were asked to answer several questions about the trial and each target instance in the image, regardless of whether they had selected the instance or not. Prior to the search task, participants saw the same training trials as in Experiment 1.

#### Ratings of Trial-Level Features and Difficulty

Following the search task, participants rated the clarity of the resolution of the object in the search template (1 = not at all, 5 = extremely clear), how confident they were that they knew what the object in the search template was (1 = not at all confident, 5 = extremely confident), and the color of the object in the search template (11 options plus unknown). Additionally, participants rated how difficult it was to find objects that were the same type as the search template (1 = not at all difficult, 5 = extremely difficult).

#### Ratings of Target Instance-Level Features

**Figure 5:**
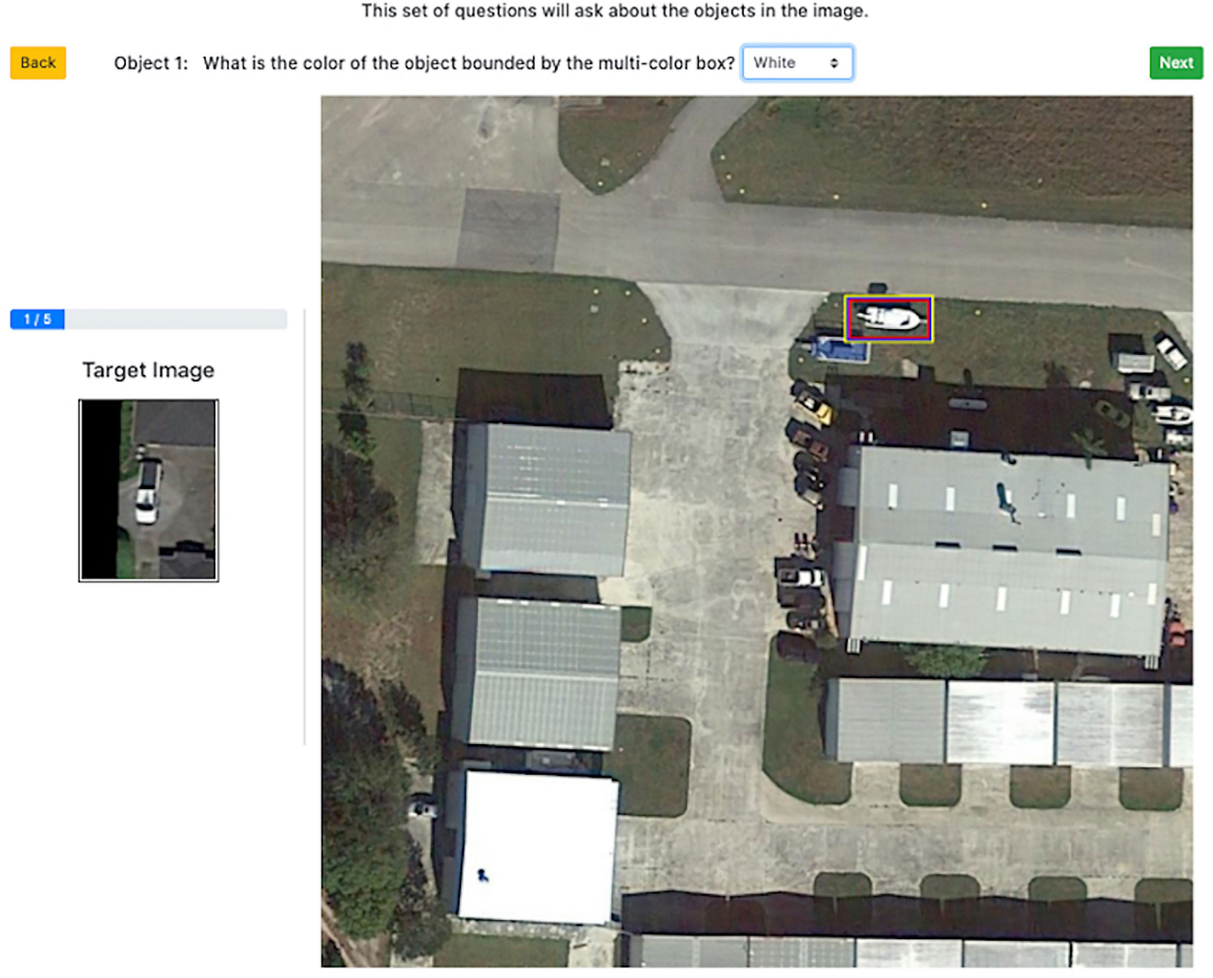
Screenshot of instance-level color rating during Experiment 2 trial. Image provided by iSAID dataset (Zamir et al., 2019) using Google Earth images.

Next participants saw a series of questions for each ground truth target instance in the aerial search image. The same set of target instances were shown to all participants regardless of which objects they selected in the first part of the task. Target instances were highlighted with a multi-colored box (colorblind friendly) for distinctiveness (Figure 5). For each target instance, participants were asked to rate how clear the resolution of the object was (1 = not at all, 5 = extremely clear), the color of the target instance (11 options plus unknown), and whether the object was the same category as the search template (1 = yes, 2 = no). For each target instance, a search template-instance color match was defined as providing the same color for both the search template and target instance (1 = yes, 0 = no).

#### Analyses

The overall true positive rate, false negative rate, and false discovery rate were calculated as described in Experiment 1. We constructed separate regression models for instance-level and trial-level features. Instance-level models predicted target instance selections based on instance clarity, instance color, instance size, distance of the instance from center, and the number of target instances in the image using a multilevel logistic regression as in Experiment 1. Trial-level features predicted performance on the whole trial using a simple linear regression and took into account the search template and average target instance clarity, the percent of target instances that matched the search template color, and the number of target instances in the trial. We also examined how target instance size and the match between the color of the search template and target instance affected the order of selections.

### Results

Overall performance was largely similar to Experiment 1. Across all trials and participants, the true positive rate was 72% and the false negative rate was 28%. The false discovery rate was 27%, indicating that participants often selected more objects than there were target instances. Similar to Experiment 1, the median time to complete a trial was 8.61 s (average = 17.62 s). The median time of the first selection was 6.07 s (mean = 14.14) and the median time participants took between selections was 960 ms (mean = 2.07 s). A correlation heatmap showing relationships between the features of the object in the search template and target instance features, performance, and difficulty can be found in the Supplementary Material.

#### Relationship Between Selections and Number of Targets

**Figure 6:**
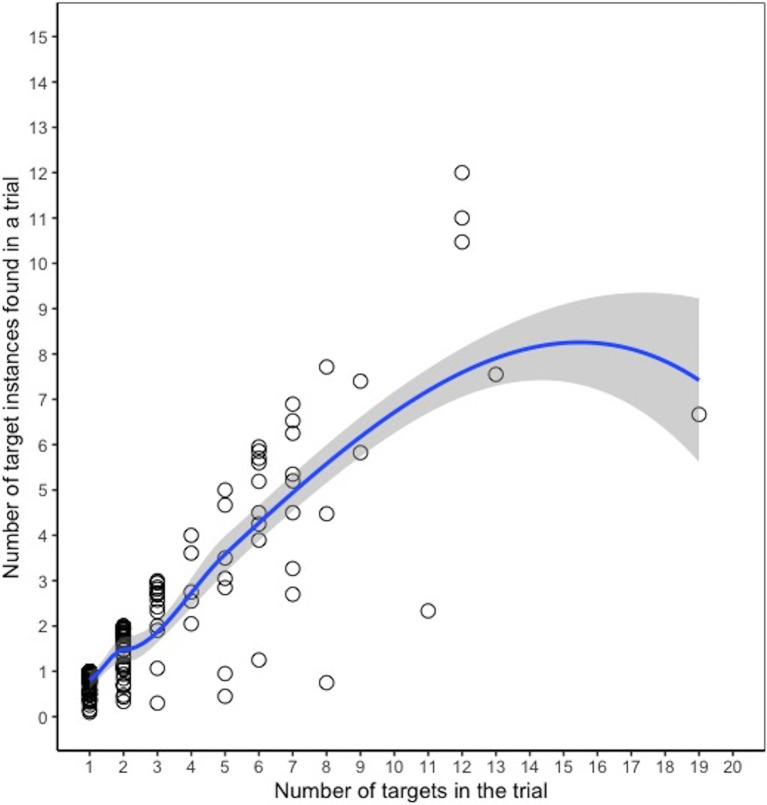
Number of targets found vs. number of targets in the trial, Experiment 2. Each dot represents a given trial, and the *y*-axis indicates the average number of targets found (across participants) in that trial. The line is a loess curve.

In 4% of trials, participants submitted it blank without selecting any objects. Among trials where an object was selected, the number of selections in each trial ranged from 1 to 55, with a median of 2 (mean = 2.67). For most trials (69%), only 1–2 objects were selected. For 29% of trials, 3–10 objects were selected. Fewer than 3% of trials had more than 10 objects selected. Consistent with Experiment 1, there was a weak positive relationship between the false negative rate for a trial and the number of targets in the trial, which was only marginally significant in this experiment [*r*(197) = 0.13, *p* = 0.07], and there was a significant negative relationship between the false positive rate and the number of targets in a trial, *r*(197) = −0.29, *p* < 0.001. A scatterplot comparing the average number of target instances found vs. the number of targets in each trial is shown in Figure 6. The number of target instances found tended to increase relatively linearly with the number of targets in the trial, with some evidence of tapering off on trials with more than 10 targets; however, as with Experiment 1, the sparse data for trials with a large number of targets makes it difficult it assess the exact point at which performance began to level off.

#### Trial-Level Models Predicting Trial Performance and Trial Difficulty

We first constructed a linear regression model for the accuracy on each trial, averaged across participants. The average true positive rate of each trial was the dependent variable, and the independent variables included average ratings of the search template clarity, the average clarity of target instances, the percentage of instances that matched the color of the search template, and the number of targets in the trial. Search template clarity, instance clarity, and color match were all significant predictors of the true positive rate of a trial; higher average clarity of instances in the trial, greater search template clarity, and a higher percentage of instances that matched the search template color were all associated with better performance on the trial (Table 4). The number of targets in the trial was a marginally significant predictor.

**Table 4:** Linear regression model predicting true positive rate from trial features, Experiment 2.

Predictor	Estimate	SE	*t*	*p*
Intercept	−0.13	0.09	−1.38	0.16
Average clarity of instances in trial	0.05	0.02	2.06	<0.05
% Of search template-instance color matches	0.21	0.04	4.24	<0.001
Search template clarity	0.17	0.02	7.16	<0.001
Number of targets	−0.01	0.01	−1.71	0.08

*R*^2^ = 0.38.

We repeated this analysis with participants’ ratings of trial difficulty as the dependent variable to examine whether the features that influenced participants’ subjective ratings of the trial were the same features that influenced their actual performance on the trial. Ratings of search template and target instance clarity were significant predictors of perceived difficulty (*B* = −0.49, *SE* = 0.05, *t* = −8.89, *p* < 0.001 and *B* = −0.62, *SE* = 0.05, *t* = −11.36, *p* < 0.001, respectively). Interestingly, the percent color match was not a significant predictor (*B* = −0.15, *SE* = 0.11, *t* = −1.36, *p* = 0.17), suggesting that participants may have been less aware of the influence that the color of the instances had on their search performance. The number of targets was also a significant negative predictor (*B* = −0.02, *SE* = 0.01, *t* = −2.11, *p* < 0.05), indicating that trials with fewer targets were perceived to be slightly more difficult. One explanation for this could be that participants had to spend more time looking for targets on these trials, which could have increased the perception of difficulty.

#### Instance-Level Model Predicting Target Instance Selections

We next constructed a multilevel logistic regression model to understand which features had the largest influence on target instance selections. Participant and trial were entered as random effects. The dependent variable was whether the target instance was selected or not (1 = selected, 0 = not selected); false positive selections were removed from this analysis, since information about object size was only available for true target instances. The fixed effects included: participants’ ratings of target instance clarity, the match between the color of the target instance and that of the search template (1 = match, 0 = not a match), the size of the target instance, the distance of the target instance from the center of the image, and the number of target instances in the image.

Almost all of the features were significant predictors of target instance selections (Table 5), with the exception of the number of targets. Target instances that were larger, rated as being clearer, and rated as being the same color as the target, and closer to the center of the image were more likely to be selected. In contrast, target instances that were rated as being less clear, a different color from the target, smaller in scale, and further from the center were more likely to be missed. There was a marginally significant effect of the number of targets, suggesting that fewer targets in the image resulted in a slightly higher likelihood of correctly identifying any given target instance in that image. The variance of the random effects was 1.06 for worker ID and 2.81 for trial, indicating that trial-related variance was greater than worker-related variance. We again examined the relative influence of each significant fixed effect by comparing the pseudo-*R*^2^ of a sequence of random effects models with single fixed effects against a baseline random effects model with no fixed effects. The target instance size variable explained the largest amount of variance (13%), suggesting this feature had the largest impact on performance, followed by clarity which explained 6% and the color match which explained 1%. In the full model with all fixed effects, the fixed effects explained 18% of the variance and the combination of fixed and random effects explained about 54%.

**Table 5:** Multilevel logistic regression model predicting accuracy from target instance features, Experiment 2.

Predictor	Estimate	SE	*z*	*p*
Intercept	−4.02	0.54	−7.51	<0.001
Target instance clarity	0.57	0.04	15.81	<0.001
Search template-instance color: match	0.53	0.08	6.30	<0.001
Target instance distance from center	−0.003	0.00	−6.89	<0.001
Log (target instance size)	0.55	0.05	10.59	<0.001
Number of targets	−0.09	0.05	−1.87	0.06

Given that the target instance clarity and size may be closely related, we examined correlations between these features to assess possible collinearity. As expected, target instance clarity was significantly correlated with target instance size (*r_s_* = 0.33, *p* < 0.001), but not excessively so. The maximum variance inflation factor (VIF) for the model was 1.06, suggesting no evidence of multicollinearity among the predictor variables.

Table 6 shows the mean or median values for the significant predictors across all true positive selections and all false negatives. Since the search template-instance color match variable was dichotomous, this table reports the percentage of true positives and false negatives that were rated as being a color match.

**Table 6:** Average target instance features by accuracy, Experiment 2.

Accuracy	Average target instance clarity	% Search template-instance color match	Average target instance size (approx.)	Average distance from center
False negative	3.02	31	81 × 81	327
True positive	3.88	45	142 × 142	293

The average target instance size is approximate and is based on the square root of the average area (in pixels) of the target instance. The dimension of the image was 800 × 800 pixels.

**Figure 7:**
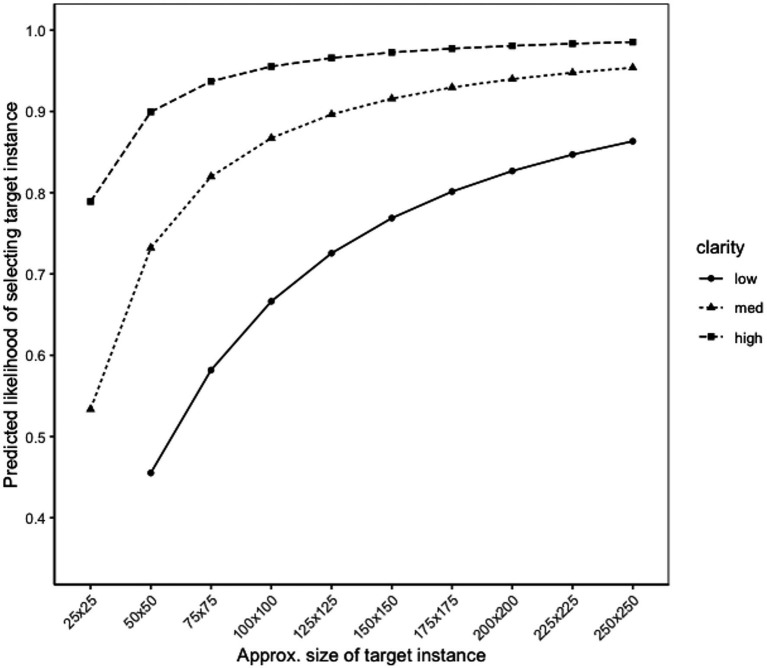
Likelihood of identifying target instance, by size and clarity.

Figure 7 shows the predicted probabilities of correctly selecting a target instance (i.e., probability of a true positive) from a logistic regression model based on size and clarity. Similar to Experiment 1, the median size of the target instances in this experiment was approximately 65 × 65 pixels in total area. Figure 7 suggests that performance can still be quite high with small target instances if their clarity is high. With low clarity target instances, the size needs to be significantly greater in total area before the true positive rate would reach 80%.

We constructed a model to predict false positives similar to Experiment 1, with distance of the selected object from the center of the image and the number of targets in the trial entered as independent variables. Size, color, and clarity information was only available for true targets, so these were not included in the model. Consistent with Experiment 1, the fewer the number of targets in the image, the more likely a selection would be a false positive (*B* = −0.30, *SE* = 0.06, *t* = −5.36, *p* < 0.001). Trials with only one or two targets again had some of the highest false discovery rates (46 and 29%, respectively). Distance of the selected object from center was not a significant predictor in this model (*B* = −0.001, *SE* = 0.0003, *z* = −1.66, *p* = 0.09).

#### Influence of Size and Color on Selection Order

Selection order analyses were conducted in a similar manner as in Experiment 1. We limited analysis to true positive selections only and selected trials with at least two target instance selections. We again limited the analysis to the first 10 selections, since the vast majority of trials had fewer than this number of selections. These criteria resulted in 5,293 true positive selections and at least 60 data points for each possible order that a target instance was selected (1:10) in its respective trial. There were 1,397 true positive selections that were selected first in their respective trials, and 66 true positive selections that were selected 10th in their respective trials. As before, target instance size within the trial was ranked in descending order (large first). We calculated the Spearman’s correlation between the ranked size of a target instance within a trial and the order in which the target instances were selected. Consistent with Experiment 1, there was a significant correlation between the ranked size and selection order (*r*_s_ = 0.45, *p* < 0.001). The correlation between selection order and arbitrary ranks was *r*_s_ = 0.36 (*p* < 0.001), which was significantly lower than the correlation between selection order and ranked size (Williams’ *t* = −6.62, *p* < 0.001).

**Figure 8:**
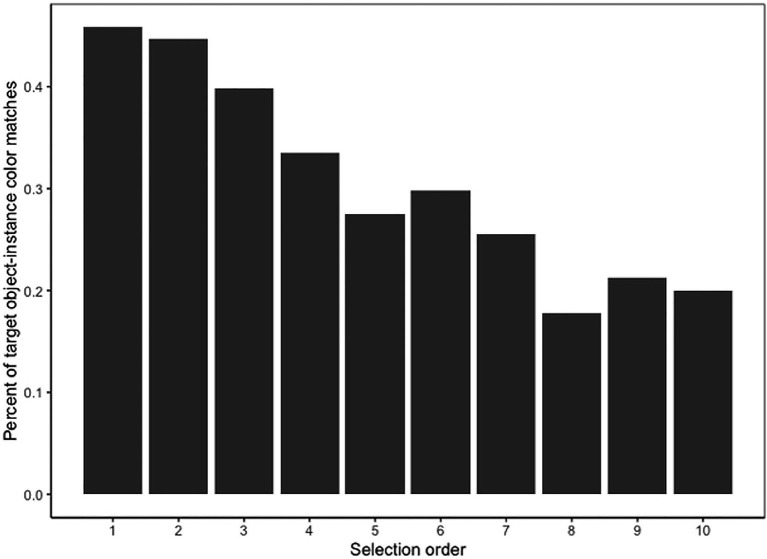
Percent of search template-instance color matches by selection order.

We also examined the relationship between the search template-instance color match and instance clarity and the order in which target instances were selected, to examine whether participants first selected target instances that matched the search template color or that had higher clarity. Since color match was dichotomous (1 = match, 0 = no match), a negative correlation would indicate that matches had rank scores that were lower in magnitude, which would suggest that participants selected matches earlier than non-matches. There was a small but statistically significant negative correlation between selection order and the search template-instance color match (*r*_s_ = −0.15, *p* < 0.001), but no correlation between selection order and instance clarity (*r*_s_ = 0.00, *p* = 0.89). Figure 8 illustrates that, among target instances that were selected first, 46% matched the color of the search template and among target instances that were selected 10th, about 20% matched the color of the search template. Moreover, among the target instances that were selected first, the average reaction time for target instances that matched the search template color was 8.69 s, while the average reaction time for target instances that did not match the search template color was 11.13 s. This pattern suggests that target instance color played a role in guiding participants’ visual search.

### Discussion

Experiment 2 replicated findings from Experiment 1 and examined how additional image features impact performance and search strategies in an aerial photograph visual search task. Experiment 2 again found that the task was fairly challenging for participants and that several features of target instances influenced performance, including the size, clarity, location, and color of the target instances. Consistent with Experiment 1, the size of the target instance had the largest effect on performance, with smaller target instances less likely to be selected. The number of targets in the trial did not have a significant effect on the likelihood of identifying a target instance, but it did have a significant effect on the likelihood of selecting additional non-target instances, suggesting that this was more likely when there was a small number of true targets in the trial. Other features of target instances had smaller but significant effects on accuracy. Target instances that were correctly selected tended to have higher perceived clarity and were more likely to match the color of the search template, compared to target instances that were missed. Target instance size and the match between the color of the target instance and search template influenced participants’ search strategies, as indicated by the order in which target instances were selected. Target instances that were smaller in size or that appeared to differ from the color of the search template tended to be selected later in the trial, suggesting that these target instances were less salient to participants. Interestingly, the match between the target instance and search template was not a significant factor for how difficult participants found the trial, suggesting that participants may be less aware of the influence of color than target instance clarity, which was the primary influencer of trial difficulty ratings.

## Overall Discussion

Aerial images can be challenging to search due to the unfamiliar viewpoints and scales of objects, along with potentially low resolution and degraded information about color. The present study examined an aerial image visual search task for which machine learning approaches are currently being developed and identified several image features that impact human performance and search strategies. Previous research has identified size, color, and orientation as some of the most important factors that guide attention in classic visual search tasks (Wolfe and Horowitz, 2017). The present study demonstrated how size, color, distance from center, clarity, and number of targets influence visual search performance in aerial image search tasks, when participants are simply prompted to look for all instances of an object class in an aerial photograph.

Visual similarity between the search template and the objects likely played a role in the task used in this study (Duncan and Humphreys, 1989). While this study was not designed to disentangle the role of similarity from the image features, future research could identify features that influence performance without relying on similarity. For example, providing a class label (e.g., “car”) instead of a search template image could help clarify which features influence performance when participants are unable to look for the visual similarity between the search template image and targets. For the purpose of the present study, we chose to use visual search templates, since the iSAID and DOTA image datasets are commonly used for training machine learning algorithms, and we wanted to assess human performance on a similar task to help enable fair machine-human comparisons for future efforts that use these datasets.

Task ambiguity is an important design consideration for the kinds of tasks used in this study. For example, the prompt for participants did not specify what counted as being the same “type” as the search template. We expected there to be numerous cases, where participants could be unclear on what counted as belonging to the same type, such as when the search template was a sedan and there were instances of sedans, trucks, vans, and other types of moving vehicles in the larger search image. Similarly, we expected there to be cases where the search template itself was ambiguous and participants may have been unsure what type of object they were searching for. Since the use case of interest for the present study was search of aerial photographs, it was a reasonable tradeoff to allow task ambiguity in order to study how people use image features in these real-world tasks. Additionally, while the current study was focused primarily on identifying what features predict the correct identification of a target, future research should explore what object features make false positives more likely. A limitation of the present study was the lack of feature information (e.g., color, clarity, etc.) about false positive selections, making it difficult to address this question. Nevertheless, understanding when humans are more likely to make a false positive judgment is also important for understanding the limitations of human performance.

It is also worth noting that, while the current study focused on establishing a benchmark of typical human performance, future research could examine how expert performance differs on this type of real-world search task. The task used in the current study did not require expertise since images of the search templates were always provided. However, some aerial search tasks could require more training or expertise, especially if a search template image is not provided, and for these tasks a separate benchmark for experts would be more suitable to use as a basis for comparison for machine learning algorithms.

As machine learning approaches become more common for complex, real-world visual search tasks, it is important to understand how humans perform on these tasks so that machine learning approaches can be evaluated against human benchmarks and to understand when machine learning approaches might offer the most benefit. The present study suggests that machine learning approaches to geospatial analysis could focus on mastering performance on images that contain very small target instances, images that contain wide variation in color between the search template and instance, and images that have poor resolution resulting in low visual clarity of the image. Some human-machine teaming paradigms have machines perform initial analyses rapidly (leveraging the strength of repetition without fatigue) while humans provide verification (Arthur et al., 2020). Understanding how various image features impact human performance on these real-world tasks can help identify which images may be more challenging for humans and could benefit from being processed by machines first. Future work should continue to examine human performance on complex, real-world visual search tasks and the features that influence their search strategies in order to understand how to create effective human-machine partnerships.

## Data Availability Statement

The datasets presented in this study can be found in online repositories. The names of the repository/repositories and accession number(s) can be found at: https://osf.io/kzgbq/?view_only=5bb6072e00a44a7084e32b523c50a000.

## Ethics Statement

The studies involving human participants were reviewed and approved by Environmental Health and Safety Group at the Johns Hopkins University Applied Physics Laboratory. The patients/participants provided their written informed consent to participate in this study.

## Author Contributions

RR, HC, and ND contributed to experiment design, analysis, and drafting of the manuscript. JH developed the web app used for experimentation and contributed to experiment design. WG-R and BW provided contributions to experiment design and critical reviews of the manuscript. All authors contributed to the article and approved the submitted version.

## Funding

This work was supported by the Office of the Director of National Intelligence (ODNI), Intelligence Advanced Research Projects Activity (IARPA), *via* IARPA contract no. 2017-17032700004-005 under the MICrONS program.

## Author Disclaimer

The views and conclusions contained herein are those of the authors and should not be interpreted as necessarily representing the official policies or endorsements, either expressed or implied, of the ODNI, IARPA, or the U.S. Government. The U.S. Government is authorized to reproduce and distribute reprints for Governmental purposes notwithstanding any copyright annotation therein.

## Conflict of Interest

The authors declare that the research was conducted in the absence of any commercial or financial relationships that could be construed as a potential conflict of interest.

## Publisher’s Note

All claims expressed in this article are solely those of the authors and do not necessarily represent those of their affiliated organizations, or those of the publisher, the editors and the reviewers. Any product that may be evaluated in this article, or claim that may be made by its manufacturer, is not guaranteed or endorsed by the publisher.
