# Supplementary material for: Benchmarking Human Performance for Visual Search of Aerial Images
Source: Front Psychol. 2021 Dec 14;12:733021. doi: 10.3389/fpsyg.2021.733021 (PMC8713551; doi:10.3389/fpsyg.2021.733021)
Supplement: Supplementary file 1 [file Data_Sheet_1.PDF]

Supplementary Material

1 Experiment 1

1.1 Training Trials

Participants completed three training trials before the experimental trials. A goal of the training was to teach participants that objects need not be the same color or size of the target example in order to be considered a match. The training trials presented participants with a matching scenario where they had to correctly identify instances of the target object within a simplified scene consisting of basic shapes of various sizes and colors (Supplementary Figure 1). Participants were presented with a black target object in the left panel and were asked to select objects that were the same type as the target. Participants received immediate feedback on their selections.

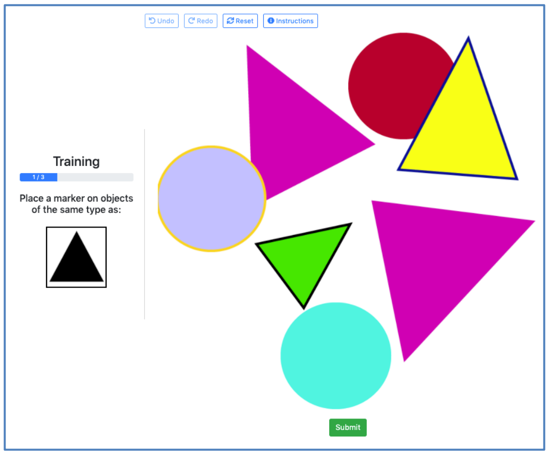

Supplementary Figure 1. Example of a training trial in Experiment 1.

1.2 Aerial Search Images

The resolution of the original images from the DOTA dataset ranged from 800 x 800 to 4,000 x 4,000 pixels. To maintain consistency across images, all images were tiled and scaled to 800 x 800 (using the procedure outlined by the authors of the iSAID dataset).

1.3 Search Templates

Search templates were selected to have sufficiently distinguishing image features, unambiguous semantic labels (meaning that multiple expert annotators agreed on the label), and no requirement of prior knowledge/experience for the classes to be recognizable. The search template was sampled to meet a minimum area requirement (30 x 30 pixels) to ensure that it was large enough for participants to see. Search templates were sampled to be reasonably isolated from other entities in the image, sufficiently distanced from image edges, and sufficiently large (i.e., have a minimum area of 1,000 pixels). Finally, a bounding box with a fixed padding amount (50 pixels on each side) was created around the search template during sampling in order to provide some scene context in the thumbnail image.

1.4 Quality Monitoring Trials

Gold standard trials were included to ensure that participants were able to identify the correct objects in a simplified version of the task. Gold standard trials contained the same instructions as experimental trials but presented a collection of thumbnail images of a variety of different object classes in different sizes and orientations (Supplementary Figure 2). The objects in the thumbnails were sampled from a variety of scene images. There were three gold standard trials in each trial block, presented at fixed locations within the block (trial 2, 12, and 17). Correct answers were defined as being within +/- 3 of the true number of counts for the first and third gold standard trials, or if their response was not within +/- 3 of all reasonable answers for the second gold standard trial. Different criteria were allowed for the second gold standard trial because the target image (car) was more ambiguous, and participants may have identified small vehicles, large vehicles, or both. Data associated with participants who did not correctly answer gold standard trials was not included in the analysis.

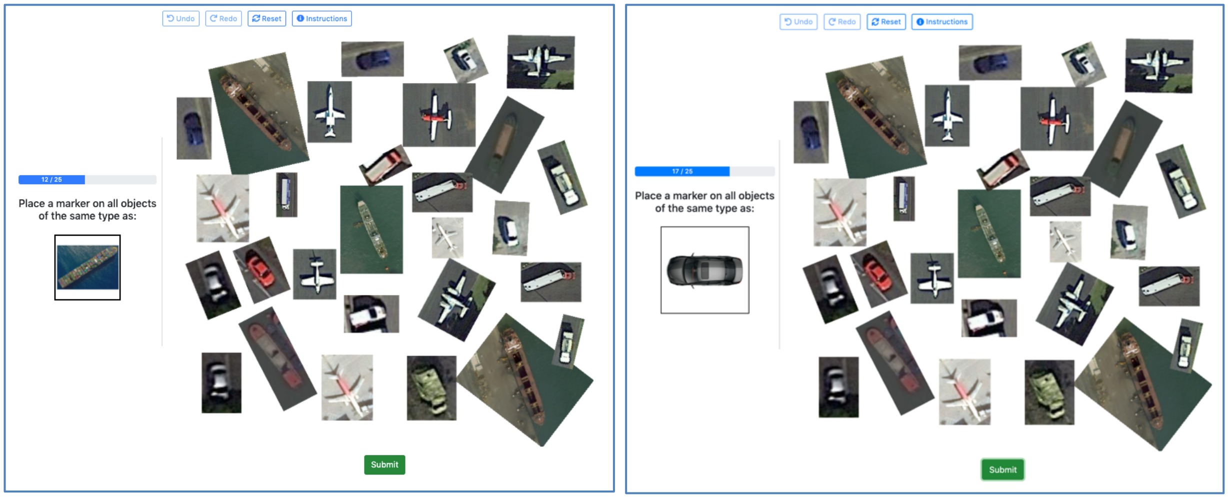

Supplementary Figure 2. Examples of gold standard trials in Experiment 1. The image on the right shows an example of the gold standard trial with the target image of a car which had different scoring criteria due to more ambiguity.

1.5 Post-Task Survey

A post-task survey was administered to better understand participants' experiences during the task and the features that affected the perceived difficulty of the trials. Participants were asked how enjoyable (1 = not at all enjoyable, 5 = extremely enjoyable) and difficult (1 = extremely difficult, 5 = extremely easy) the task was, and were asked to complete an adapted version of the NASA-TLX (Hart & Staveland, 1988). The adapted NASA-TLX survey asked participants to rate the mental demand, physical demand, frustration, hurriedness, level of effort, and level of success experienced during the task on scales of 1-7 (1 = very low/not at all, 7 = very high). Finally, participants were asked to answer open-ended questions about which features of the image made it easier or harder to find the targets.

1.6 Post-Task Survey Results

Most (67%) participants reported that the task was very or extremely enjoyable; 27% of participants reported that the task was somewhat or extremely difficult and 48% reported that the task was somewhat or extremely easy. The average overall raw NASA-TLX score was 3.90 (SD = 1.62), indicating a medium subjective workload. The average rating for level of effort was 5.91 (SD = 1.33), indicating participants spent a fairly high level of effort on the task. Participants' feedback on the features that made the task more or less difficult were analyzed by looking at the most common positive and negative words in participants' open-ended responses using the tidytext package in R. The vast majority of words identified were related to object clarity. When asked which features made the task easier, the most common positive word mentioned was "clear" (mentioned 63% of the time a positive word was identified). When asked which features made the task more difficult, the most common negative word mentioned was "blurry" (mentioned 45% of the time a negative word was identified). These results suggest that target object clarity or target instance clarity had an impact on perceived difficulty and perhaps accuracy, a possibility we examined further in Experiment 2.

2 Experiment 2

2.1 Correlational Analysis

A correlation heatmap showing relationships between the features of the object in the search template (target object) and target instance features, performance, and difficulty is shown in Supplementary Figure 3. As expected, confidence in the target object was positively correlated with the clarity of the target object ($r_s = .63$, $p < .001$) and negatively correlated with the perceived difficulty of the trial ($r_s = -.46$, $p < .001$). Additionally, believing that a target instance matched the class of the target object was positively correlated with a true positive selection ($r_s = .60$, $p < .001$).

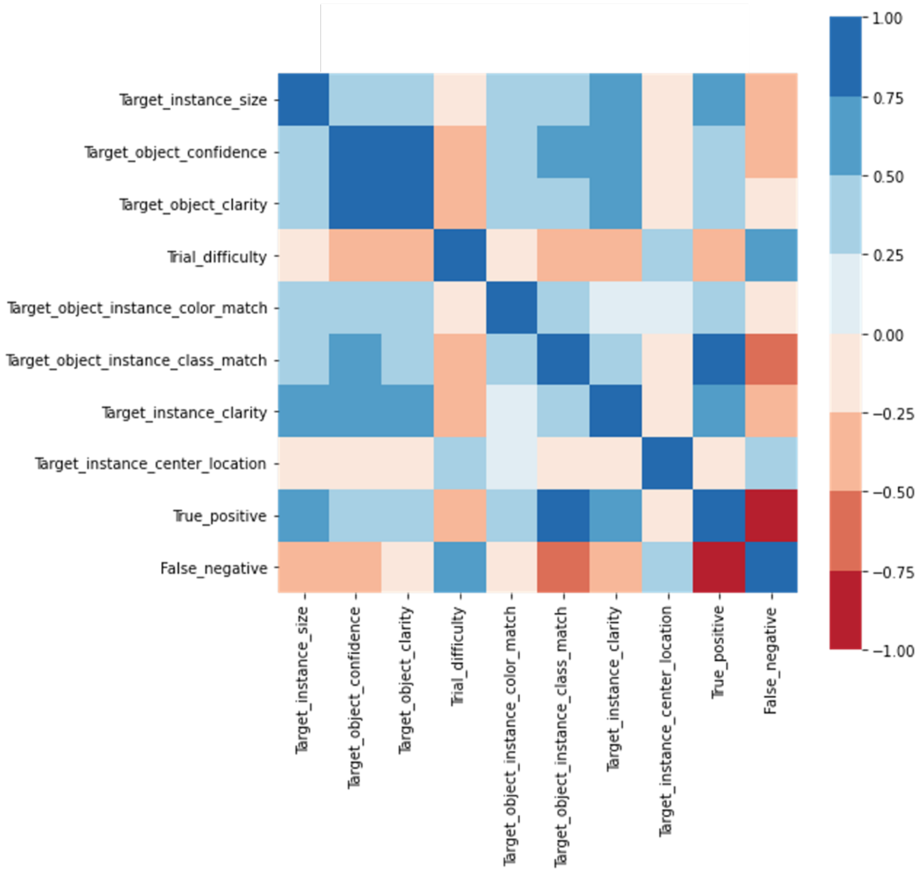

Supplementary Figure 3. Spearman correlation coefficients between search template features, target instance features and human performance.

References

Hart, S. G., & Staveland, L. E. (1988). Development of NASA-TLX (Task Load Index): Results of Empirical and Theoretical Research. In *Advances in Psychology* (Vol. 52, pp. 139–183). North-Holland.

Kuhn, H. W. (1955). The Hungarian method for the assignment problem. *Naval Research Logistics Quarterly*, *2*(1–2), 83–97. https://doi.org/10.1002/nav.3800020109
